# Emerging Evidence on the Use of Probiotics and Prebiotics to Improve the Gut Microbiota of Older Adults with Frailty Syndrome: A Narrative Review

**DOI:** 10.1007/s12603-022-1842-4

**Published:** 2022-09-17

**Authors:** B. Sánchez y Sánchez de la Barquera, B.E. Martínez Carrillo, J.F. Aguirre Garrido, R. Martínez Méndez, A.D. Benítez Arciniega, R. Valdés Ramos, Alexandra Estela Soto Piña

**Affiliations:** 1 Facultad de Medicina, Universidad Autónoma del Estado de México, Paseo Tollocan esq. Jesús Carranza, Z.C. 50180, Toluca de Lerdo, México; 2 Departamento de Ciencias Ambientales, Universidad Autónoma Metropolitana, Lerma, México; 3 Facultad de Ingeniería, Universidad Autónoma del Estado de México, Toluca, México

**Keywords:** Frailty, gut microbiota, aging, probiotics, prebiotics, inflammation

## Abstract

**Background:**

The gut microbiota can impact older adults' health, especially in patients with frailty syndrome. Understanding the association between the gut microbiota and frailty syndrome will help to explain the etiology of age-related diseases. Low-grade systemic inflammation is a factor leading to geriatric disorders, which is known as “inflammaging”. Intestinal dysbiosis has a direct relationship with low-grade systemic inflammation because when the natural gut barrier is altered by age or other factors, some microorganisms or their metabolites can cross this barrier and reach the systemic circulation.

**Objectives:**

This review had two general goals: first, to describe the characteristics of the gut microbiota associated with age-related diseases, specifically frailty syndrome. The second aim was to identify potential interventions to improve the composition and function of intestinal microbiota, consequently lessening the burden of patients with frailty syndrome.

**Methods:**

A search of scientific evidence was performed in PubMed, Science Direct, and Redalyc using keywords such as “frailty”, “elderly”, “nutrient interventions”, “probiotics”, and “prebiotics”. We included studies reporting the effects of nutrient supplementation on frailty syndrome and older adults. These studies were analyzed to identify novel therapeutic alternatives to improve gut microbiota characteristics as well as subclinical signs related to this condition.

**Results:**

The gut microbiota participates in many metabolic processes that have an impact on the brain, muscles, and other organs. These processes integrate feedback mechanisms, comprising their respective axis with the intestine and the gut microbiota. Alterations in these associations can lead to frailty. We report a few interventions that demonstrate that prebiotics and probiotics could modulate the gut microbiota in humans. Furthermore, other nutritional interventions could be used in patients with frailty syndrome.

**Conclusion:**

Probiotics and prebiotics may potentially prevent frailty syndrome or improve the quality of life of patients with this disorder. However, there is not enough information about their appropriate doses and periods of administration. Therefore, further investigations are required to determine these factors and improve their efficacy as therapeutic approaches for frailty syndrome.

## Abbreviations

*ACTH*: *Adrenocorticotropin hormone*
*BFM*: *Body fat mass*
*BMI*: *Body mass index*
*BNR17*: *Probiotic strain isolated from human breast milk*
*CCL11*: *Motif chemokine 11*
*CD4*: *Cluster of differentiation 4*
*CD8*: *Cluster of differentiation 8*
*CFUs*: *Colony Forming Units*
*COVID-19*: *Coronavirus disease 2019*
*CXCL11*: *Motif chemokine ligand 11*
*d*: *Day*
*FBS*: *Fasting Blood Sugar*
*GI*: *Gastrointestinal tract*
*hCRP*: *Hgh sensitivity C Reactive Protein*
*HPA*: *Hypothalamic-pituitary-adrenal axis*
*IFN*: *Interferon*
*IL6*: *Interleukin-6*
*IL8*: *Interleukin-8*
*IL10*: *Interleukin-10*
*IL17A*: *Interleukin-17* ^*a*^
*ISAPP*: *International Scientific Association for Probiotics and Prebiotics*
*LBP*: *Lipopolysaccharide-binding protein*
*M*: *Maintenance*
*NSP*: *Nonstarch polysaccharides*
*RS*: *Resistant starch*
*SARS-CoV-2*: *Severe acute respiratory syndrome coronavirus 2 of the genus Betacoronavirus*
*SCF*: *Soluble corn fiber*
*SCFAs*: *Short-chain fatty acids*
*TNF*: *Tumor necrosis factor*
*WG*: *Whole grain*
*WL*: *Weight loss*.

## Introduction

**F**railty syndrome is a condition that develops as people age, and the gastrointestinal (GI) tract plays a critical role in its development. The GI tract is responsible not only for digestion and absorption but also for the acquisition of food immune tolerance and the habitat of commensal microorganisms (microbiota) (1). The gut microbiota changes throughout the human lifespan, and it exerts an impact on health, especially during the aging process. The intestinal microbiota is a community that includes more than 100 billion microorganisms, with a unique conformation for each individual, including bacteria, viruses, and yeast (2). Some bacterial phyla commonly found in the human intestinal microbiota are *Proteobacteria, Verrucomicrobia, Actinobacteria, Fusobacteria Bacteroidetes*, and *Firmicutes. Bacteroidetes* and *Firmicutes* represent approximately 90% of the total microbiota in humans (3). The gut microbiota composition and diversity can change through the aging process and can influence optimal immune system performance, which is essential to prevent the development of age-related diseases (Rinninella et al., 2019, Hasan and Yang, 2019).

The biological causes of some age-related diseases are currently known; one of them involves changes in the gut microbiota. However, many aspects of the relationship between microbiota and frailty remain unclear and need to be further investigated. This is required to improve or identify new interventions to slow the progression of frailty syndrome and its consequences, thereby contributing to a better quality of life during aging.

Organisms undergo transformation processes and cellular changes from conception until death; aging is one of these stages (5). Michael R. Rose (6) defines aging as “a persistent decrease in the state of health dependent on the specific age of an organism due to internal physiological deterioration”. This definition was modified to change the concept of “decreasing health status” into a “disarrangement process” (7). Furthermore, the World Health Organization (WHO) defines aging as “the accumulation of a great variety of molecular and cellular alterations over time, which leads to a gradual decrease in physical capacities and mental disorders, an increased risk of disease, and ultimately death” (8).

The normal course and evolution of aging are different for each individual because the conditions in which each individual reaches this stage vary, and so does the way the individual responds to them (5). There are multiple geriatric syndromes, such as the risk of falls, incontinence, delirium, or functional impairment (Bird et al., 2013, Carlson et al., 2015). They all present a set of signs and symptoms of multifactorial origin. Nonetheless, they share elements associated with aging and can trigger disability or dependency (10). Frailty syndrome is a geriatric condition that causes remarkable functional impairment; it also involves increased vulnerability and an increased risk of developing adverse health events, such as dependency, disability, hospitalization, or death, when these individuals are exposed to stressors (11). The development of frailty syndrome is mainly related to the impairment of multiple systems. Thus, an evaluation of the set of symptoms, signs, and biomarkers must be performed to diagnose it (12). Furthermore, this syndrome often coexists with other pathologies and results in unfavorable physiological consequences, requiring multiple intervention strategies (13).

Frailty syndrome is mainly of multifactorial origin and is rarely attributed to a unique cause. Some factors associated with frailty syndrome include the accumulation of cellular damage, malnutrition, sarcopenia, the deterioration of multiple systems, psychological alterations, polypharmacy, sociodemographic factors, preexisting diseases, low physical activity, and uncontrolled inflammation (14). The latter plays a major role in aging; there is a type of systemic, chronic, and low-grade inflammation produced by the continuous accumulation of antigenic load and stress, which is known as “inflammaging” (1). There is a hypothesis that endogenous cellular debris acts as the main aversive stimulus of inflammaging; therefore, it is considered an autoimmune disorder; indeed, it is also called “garbaging” (15), which functions as an accelerator of the aging process.

### Probiotics and prebiotics

The International Scientific Association for Probiotics and Prebiotics (ISAPP) gathered an expert panel to discuss themes related to probiotics and prebiotics. According to the ISAPP, a probiotic is a “live microorganism that, when administered in adequate amounts, confers a health benefit on the host” (16). Probiotics may include live microorganisms in food or supplementation, with or without a specific health claim or a probiotic drug (17). They can have different routes of administration, effect target sites, and host species targets (18). Microbial components, microbial products and dead microbes are not considered in the classification of the probiotic type; this is because there is not enough evidence about their benefits on health and safety regarding their intended use. The difference between probiotics and microbiota lies in the fact that all commensal microorganisms are isolated, characterized, and proven to have beneficial effects on health (16).

Moreover, a prebiotic is a compound that is primarily derived from vegetables or fruits, but it can also be synthetic. A prebiotic is an insoluble carbohydrate that is unable to be digested and absorbed, but it serves as an energy source for the intestinal microbiota (19). In 2016, the ISAPP discussed the definition of a prebiotic and concluded that a prebiotic is a “substrate that is selectively utilized by a host microorganism, conferring a health benefit” (20).

Twenty-five years ago, the term “symbiotic” was introduced to refer to a combination of a probiotic and a prebiotic (21). In 2019, the ISAPP updated the definition of symbiotic to “a mixture comprising live microorganisms and substrate(s) selectively utilized by host microorganisms that confers a health benefit on the host” (22). There are two subcategories of symbiotics: first, complementary symbiotics, which refers to symbiotics designed to target autochthonous microorganisms, and second, synergistic symbiotics, in which the substrates are designed to be used selectively by the coadministered microorganisms (23). Symbiotics are not confined to human applications and can be applied to intestinal and extraintestinal microorganisms, but their beneficial effects on health must be confirmed (22).

### Review goals

The association between frailty syndrome and the gut microbiota is complex, and it has generated interest among researchers to focus their efforts on identifying the suitable consumption of certain foods to improve microbiota diversity. In this way, we can better explain how the quality of a diet can positively or negatively affect the intestinal microbiota and the development of frailty. Certain dietary patterns favor a microbiota composition that could be beneficial for health. For example, the consumption of ultra-processed foods is associated with decreased muscle mass and strength, which are two main characteristics of frailty syndrome (24). Additionally, it can negatively affect the microbiome, specifically related to proinflammatory processes (25). Therefore, it is important to highlight the complex relationships between modifiable factors (such as diet and physical activity) in the search for nutrients that prevent or limit this disease (26).

The main goal of this review was to show evidence that supports the relationship between the gut microbiota of older adults and frailty syndrome. Furthermore, it is necessary to determine what kind of modifications in the gut microbiota can cause frailty or can be part of the approaches for its treatment or prevention.

The specific aims of this review are listed below:

a. To describe the characteristics of the intestinal microbiota that can lead to age-related diseases, specifically frailty syndrome.
b. To identify potential interventions that improve intestinal microbiota composition and diversity, which can improve the quality of life of patients with frailty syndrome.

## Methodology

### Search strategy criteria

This was a narrative review where the selection criteria included systematic reviews, experimental designs, intervention designs, and clinical studies. The obtained information was organized according to the research aims. After organization, the information was analyzed and synthesized to finally draw a conclusion. This analysis provides current evidence about the effects of probiotics, prebiotics, and other types of nutritional supplementation to modulate the intestinal microbiota as well as their potential benefits in preventing or treating frailty syndrome.

We followed the methodology of a Narrative Review, considering the Scale for the Assessment of Narrative Review Articles (SANRA). We screened three search engines (PubMed, Science Direct, and Redalyc). The following keywords were used in the search strategy: (aging* AND frailty), (gut microbiota* OR microbiome* AND body composition), (brain-gut-microbiota axis), (muscle-gut-microbiota axis), (inflammation* OR inflammaging AND prebiotics* OR probiotics), (life expectancy* AND quality of life*), (dietetic *OR interventions) AND (frailty* AND elderly OR successful aging). The selection, analysis, and organization were performed by a single person, but the final manuscript was revised and approved by all the authors of this manuscript.

### Inclusion and exclusion criteria

**Figure 1:**
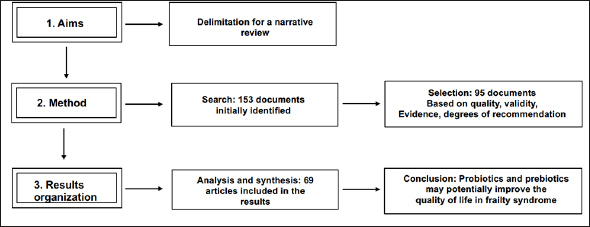
Flow chart of the methodological process of this narrative review

The inclusion criteria for the selected articles were 1) articles from the last 5 years (2017–2021), 2) articles with content following the keywords, and 3) articles in line with the specific aims of this study. However, some articles dated before 2017 were also included because of their theoretical value. Indexed journals with indicators of quality, information validity, levels of evidence, and degrees of recommendation were included as well. The excluded articles were those that were not related to the context and specific aims of this review. Additionally, articles written in any language other than English or Spanish were excluded. Articles that did not meet the inclusion criteria were excluded. A total of 153 articles were found after an initial general selection process, but 58 manuscripts were excluded because they did not focus on the main objectives of this review. Ultimately, we identified 95 articles, including reviews, meta-analyses, trials, and cohort studies, that met the inclusion criteria. From these 95 articles, 26 were included in the Introduction section and 69 were included in the Results section. Figure 1 shows the flow diagram of the article selection process.

### Data extraction and analysis from the included articles

The following data were extracted from all studies: author, year, experimental model, assessment methodology, intervention, endpoints, outcome measures (frailty conditions, description of gut microbiota, and relationships), and key findings. For clinical studies, the extracted data included the following: author, year, demographics (age and sex), sample size, intervention, follow-up duration, assessment methodology, outcome measures (frailty conditions, description of gut microbiota, and relationships), and key findings.

### Organization of the information

Every manuscript was analyzed, and the information obtained was synthesized and written to accomplish the aims of this review.

## Results

### Gut microbiota in aging and frailty

In humans, the gut microbiota is composed of 90% of the bacterial phyla *Firmicutes* and *Bacteroidetes;* the remaining 10% comprises *Actinobacteria, Proteobacteria, Fusobacteria*, and *Verrucomicrobia* (3). The gut microbiota diversity and its characterization are complex, and the bacteria are grouped into three different microbial metagenomic groups called enterotypes. The genera *Bacteroides* from the *Bacteroidaceae* family, *Prevotella* from the *Prevotellaceae* family (from the *Bacteroidetes* phylum) and *Ruminococcus* from the *Ruminococcaceae* family (from the *Firmicutes* phylum) are the most abundant genera found in these enterotypes (27). There are similarities in the proportions of these enterotypes that constitute the main microbiota according to the age group: young adults (22–48 years), older adults (65–75), centenarians (99–104 years), and semi-supercentenarians (105–109 years) (28).

This composition may vary depending on some host-related factors, such as diet, antibiotic use, age (from gestation), type of birth, lactation method, anatomical area (small intestine or colon), body mass index, exercise frequency, and intra- and extraintestinal diseases. Some of these conditions are pertinent to the host's individual characteristics, and others are not. For example, the host genetic characteristics, the morphology of the epithelium, and its immune components are individual factors that modulate the gut microbiota (29). Moreover, exposure to environmental compounds, the use of probiotics or prebiotics, and fecal transplantation are external factors that can also modify the composition of the gut microbiota (4).

Age is a determining factor in the composition (diversity) of the intestinal microbiota (O'Toole and Jeffery, 2015, O'Toole and Jeffery, 2018), and the three enterotypes of the main microbiota become less abundant as aging proceeds (28). For example, adults over 70 years old show a decrease in *Bifidobacterium* and *Clostridium* but an increase in Proteobacteria (3). The changes in the composition and functionality of the gut microbiota can result in significant alterations in the physiology of the host (32). Ticinesi and collaborators (33) showed that the bacterial taxa associated with frailty syndrome include the following: *Prevotella, Ruminococcus, Alistipes, Oscillibacter, Eubacterium, Eggerthella, Faecalibacterium, Coprobacillus, Porphyromonas, Peptococcus, Fonticella, Clostridium cluster XIVa, Lachnospiraceae, Lactobacillus, Blautia, Odoribacter, Actinomyces* and *Veillonella.* Interestingly, they also revealed that frailty was inversely associated with the diversity and relative abundance of several microbiota species.

The intestinal microbiota is responsible for nondigestive monosaccharide fermentation, producing mainly short-chain fatty acids (SCFAs), such as acetate, propionate, and butyrate (34). A certain amount of SCFAs absorbs from the intestine into the systemic circulation and is capable of producing effects at different levels: from local modifications in the intestine (improving mucus production and regulating permeability) to the regulation of energy metabolism (35). SCFAs have a remarkable effect on the immune system (36). Butyrate may modulate inflammation by differentiating regulatory T lymphocytes (37). In addition, limited microbiota diversity and SCFA receptor deficiency affect microglial function (38).

**Figure 2:**
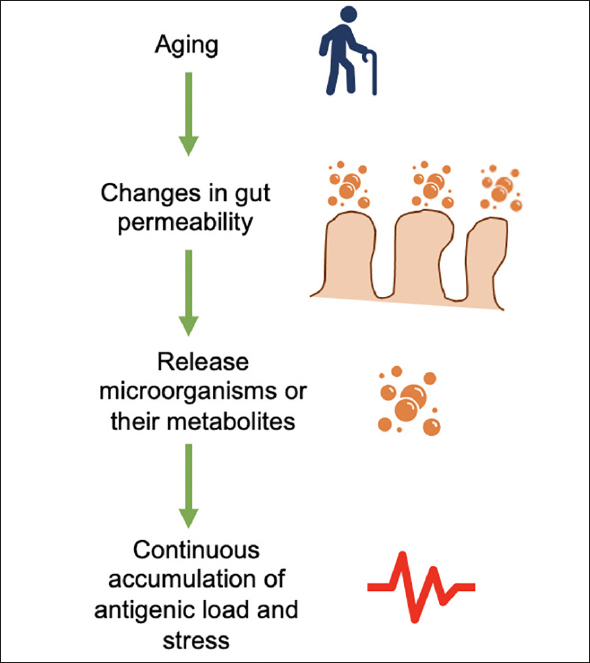
Age-related changes in the intestinal tract lead to low-grade chronic inflammation, with increased gut permeability due to age, and microorganisms or their metabolites are released into the blood with a consequent accumulation of antigenic load

Changes in intestinal permeability are typical of aging and allow the release of microorganisms or their metabolites into the circulation. In this way, the immune system is activated to secrete mediators of inflammation. Therefore, the microbiota has a fundamental role in developing chronic inflammation (Figure 2) (Di Sabatino et al., 2018, Komanduri et al., 2019). For instance, zonulin, a protein that modulates gut permeability, is significantly decreased in older adults with frailty, and the microbiota has a close association with gut permeability and inflammation (40).

The brain-intestine axis, with the participation of the intestinal microbiome, has an important influence on aging, but this can also impact the gut microbiota (41). This indicates that the relationship of the brain-intestine axis with aging is bidirectional. For example, changes in the gut microbiota can trigger cognitive impairment (42). In the opposite sense, neurodegenerative diseases with an accumulation of beta-amyloid peptides can lead to generalized inflammation and changes in the conformation of the gut microbiota (3). Serena Verdi and collaborators demonstrated that there was a negative association between microbiota and decision-making speed and speech fluency in adults >40 years old (43). Another study showed a negative association between the relative abundance of Enterobacteriaceae and Porphyromonadaceae families and cognitive performance (44). Thus, aging affects the composition of the gut microbiota, while changes in the microbiota can accelerate age-related alterations. Moreover, gut dysbiosis and the release of inflammatory mediators promote the development of age-related pathologies (45).

**Figure 3:**
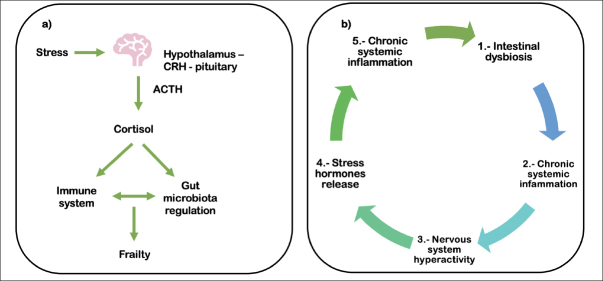
Brain-gut-microbiota axis: the relationship with the HPA axis. a) The hypothalamus, through corticotropin-releasing hormone (CRH), stimulates the pituitary gland to secrete adrenocorticotropin hormone (ACTH), which in turn stimulates the adrenal gland to secrete cortisol. Cortisol has systemic effects affecting the regulation of the gut microbiota. b) Intestinal dysbiosis may lead to chronic systemic inflammation, causing hyperactivity of the nervous system and the consequent release of stress hormones, which in turn could also induce chronic systemic inflammation and dysbiosis

The gut microbiota reacts to stress stimuli, which may alter the immune response. In aging, there are neuroendocrine changes due to stress exposure (46). In particular, hypothalamic-pituitary-adrenal (HPA) axis activity changes as people age, which is usually observed as circadian modifications in cortisol levels (47). Alterations in cortisol and adrenocorticotropin hormone (ACTH) responses related to frailty syndrome support the role of the HPA axis in this disease (Figure 3a) (Le et al., 2021, Marcos-Perez et al., 2019). Changes in the gut microbiota may lead to altered activation of the HPA axis with the consequent onset of systemic inflammation, as shown in Figure 3b (50–52). Moreover, cortisol modifications are linked with microbiota diversity in children (53), but this has not been clearly explained in older adults. In rodents, the microbiota can regulate the expression of genes involved in the HPA axis response to stress and intestinal biogenesis (54). Therefore, the association between plasma and salivary cortisol concentrations and the gut microbiota must be further investigated in cross-sectional and longitudinal studies of frailty.

**Figure 4:**
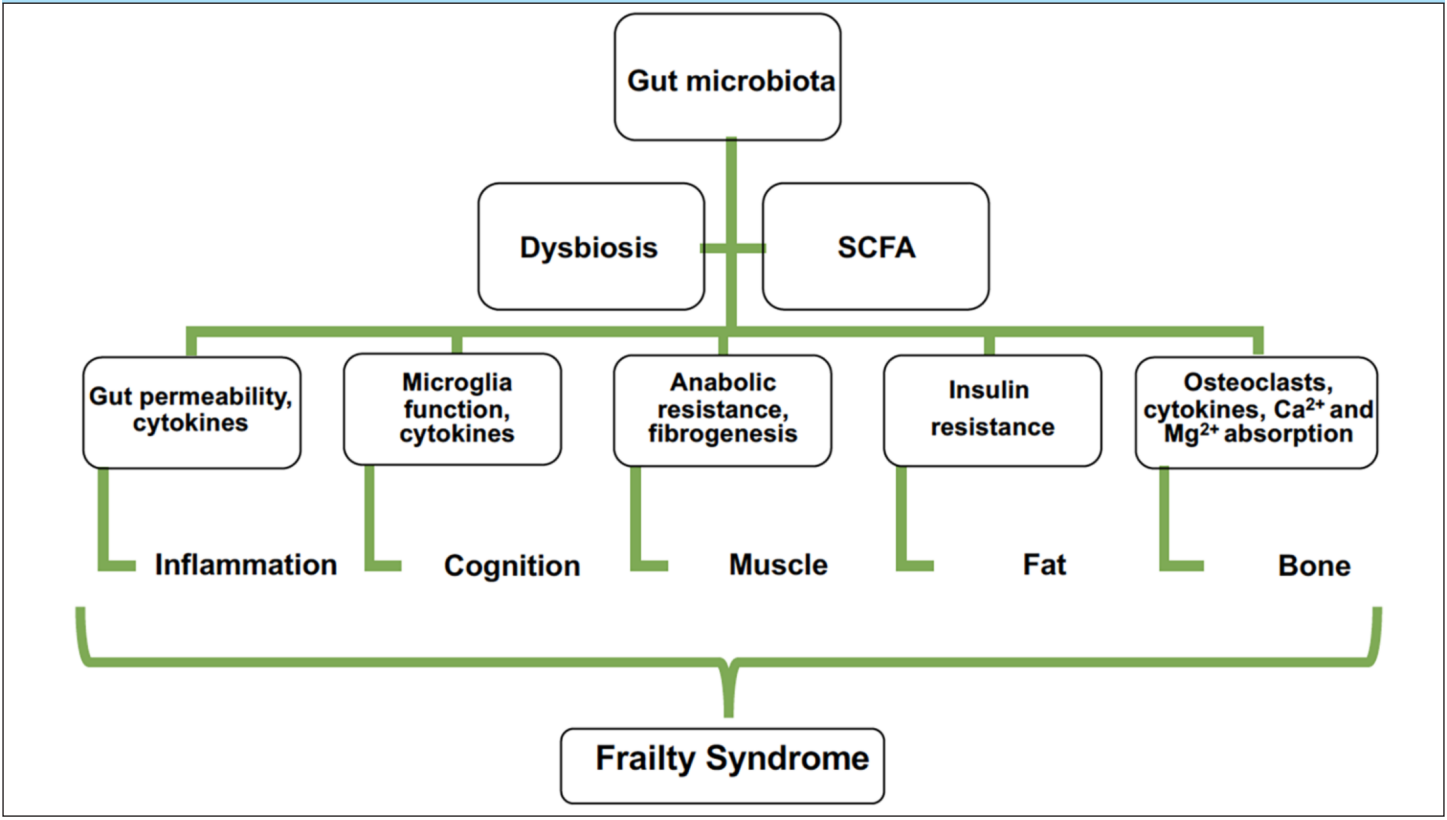
The gut microbiota may contribute to frailty onset by the impact of short-chain fatty acids (SCFAs) on the central nervous system, the promotion of inflammation, and the influence on muscle, fat, and bone, which are present in frailty syndrome either together or by themselves

The gut microbiome is involved in anabolic resistance and chronic inflammation and has direct effects on the gut barrier and the availability of proteins from the diet (55). Consequently, the presence of a gut-muscle axis is possible because of the evidence of this relationship (56). Various investigations of microbiota, body composition, and muscle strength have shown that there could be an association between the gut microbiota and frailty (33). For example, the gut microbiota is different in subjects who present with obesity from those who do not (57), which is also related to how fat is deposited in the organs (58). The production of SCFAs in older adults decreases, which is related to insulin resistance, intramuscular fat accumulation, and decreased muscle function (Poggiogalle et al., 2019, Sachs et al., 2019). This finding supports the relationship between gut dysbiosis and sarcopenic obesity. In addition, the gut microbiota is related to the onset of sarcopenia and frailty related to malnutrition (61). This involves age-related anorexia because microbiota metabolites modulate satiety and appetite through signals in the enteric nervous system (van de Wouw et al., 2017, Fetissov, 2017). Consequently, gut microbiota modifications associated with age have repercussions at different levels and are capable of triggering frailty syndrome. In addition, SCFAs influence bone metabolism, either by regulating inflammation and osteoclast activation or by modulating Ca^2+^ and Mg^2+^ absorption (63). The use of SCFAs may be a potential therapeutic option in the treatment of neurodegenerative diseases related to aging and frailty (64). Figure 4 shows how the gut microbiota may contribute to the onset of frailty syndrome.

### Treatment and possible interventions

#### Probiotics and prebiotics

Prebiotics can be included in preventive treatments, but the patients who may benefit must be previously identified (65). Additionally, prebiotics can positively influence the gut microbiota by increasing its quantity and variety, leading to an improvement in nutrient function and absorption and consequently maintaining health (66). Table 1 shows some prebiotic interventions and their doses (amount and frequency) that have generated modifications in the composition of microbiota strains in human studies. Oral administration of agave inulin increases *Bifidobacterium* in healthy adults (67), as shown in a crossover trial where it was given for periods of 21 days using 3 different doses (0 g, 5.0 g, or 7.5 g/day), with 7 days of washout between periods. The consumption of a high whole-grain diet did not lead to significant changes in the gut microbiota of healthy adults (68). Moreover, men with obesity and metabolic syndrome were subjected to four different diets for ten weeks, consisting of a maintenance diet, a nonstarchy polysaccharide diet, a resistant starch diet, and a weight loss diet, with 5.1 g, 2.5 g, 25.4 g and 2.9 g of resistant starch, respectively (69). The results show an increase in *O. guillermondii, R. bromii, S. termitis, C. leptum*, *C. cellulosi, Alistipes spp.*, and *E. rectale* and a decrease in P. cinnamivorans, microbiota diversity, acetate, propionate, and butyrate. Additionally, a crossover study included healthy adolescent females who received 0 g, 10 g, and 20 g of soluble corn fiber/day (70). The results revealed an increase in Parabacteroides in the gut microbiota.

**Table 1:** Probiotic and prebiotic interventions and their impacts on intestinal microbiota

**Type of intervention**	**Dose**	**Type of patient**	**Effect**	**Reference**
Prebiotic:				
Inulin	3 periods (0, 5.0, or 7.5 g) of agave inulin/d for 21 days, with 7 days of washouts between periods vs. placebo.	Healthy adults (BMI >18.9 — <29.5 kg/m^2^, age 20–40, free of metabolic and GI diseases).	Increase in *Bifidobacterium.*	Holscher, 2015
Whole grains	Diets high in WGs (>80 g/d) or low in WGs (<16 g/d) for 6 crossover weeks, separated by 4 washout weeks.	Healthy adults (BMI 20–35 kg/m^2^, aged 40–65 years, habitual WG consumption <24 g/day).	No significant changes.	Ampatzoglou, 2015
Resistant starch	Follow 4 different diets during a period of 10 weeks: M, NSP, RS, and WL with 5.1, 2.5, 25.4, and 2.9 g/dL of RS, respectively.	Obese, metabolic syndrome, men (BMI 27.9–51.3 kg/m^2^, age 27–73).	Increase in *Oscillospira guillermondii, R. bromii, Sporobacter termitis, Clostridium leptum, C. cellulosi, Alistipes spp, E. rectale.* Decrease in *Papillibacter cinnamivorans*, microbiota diversity, acetate, propionate, and butyrate.	Salonen, 2014
Soluble corn fiber	0, 10, and 20 g of fiber/d from SCF, 3 phases of crossover lasting 4 weeks each, with 3 weeks of washout.	Healthy adolescent females (BMI in kg/m^2^ >90th percentile for age, aged 11–14 years, identified as healthy).	Increase in *Parabacteroides.*	Whisner, 2016
Probiotic:				
Lactobacillus casei Shirota	6.5 × 10^9^ CFU/dose/d for 3 months.	Metabolic syndrome (BMI 35.4 ± 5.3 kg/m^2^, age 51.5 ± 11.4 years) healthy controls.	No influence in the LBP compared with controls.	Leber, 2012
Lactobacillus gasseri	BNR17, 1 x 10^10^ CFU/dose, 6 doses/d for 12 weeks vs. placebo.	Obese adults (BMI > 23 kg/m^2^, age 19–60, nonpregnant and FBS ≥ 100 mg/dL).	Reduced weight and waist and hip circumferences; however, there were no significant changes.	Jung, 2013
Bifidobacterium breve B-3	50 × 10^9^ CFU/dose/d for 12 weeks vs. placebo.	Overweight adults (BMI 24–30 kg/m^2^) aged 40–69 years.	Lower BFM and improved blood parameters related to liver functions and inflammation, such as c-glutamyltranspeptidase and hCRP.	Minami, 2015


d: day; BMI: body mass index; GI: gastrointestinal; WG: whole grain; RS: resistant starch; M: maintenance; NSP: nonstarch polysaccharides; WL: weight loss; SCF: soluble corn fiber; CFUs: colony-forming units; LBP: lipopolysaccharide-binding protein; BNR17: probiotic strain isolated from human breast milk; FBS: fasting blood sugar; BFM: body fat mass; hCRP: high-sensitivity C-reactive protein.

Treatment with probiotics and prebiotics may be a good strategy when trying to improve the composition of gut microbiota, as they can help to maintain normobiosis and decrease systemic inflammation (71). Probiotic interest is increasing, and it should be noted that supplementation with probiotics can be part of a treatment that involves other substances due to the comorbidity of various conditions, especially during aging (72). Table 1 shows some studies about the metabolic impact of some probiotic interventions in different populations, indicating the amount and frequency of each probiotic. Although the interventions were not performed specifically in patients with frailty syndrome, they had an impact on body composition, blood parameters, and clinical conditions. Oral administration of 6.5 x 10^9^ colony forming units (CFUs) of *Lactobacillus casei Shirota* in patients with metabolic syndrome did not influence lipopolysaccharide-binding protein (LBP) compared with controls (73). Furthermore, the oral administration of *Lactobacillus gasseri* (a probiotic strain isolated from human breast milk, BNR17) at a dose of 1 x 10^10^ CFU, 6 times per day for 12 weeks, was given to patients with obesity (74). The results showed that this probiotic reduces body weight and waist and hip circumferences. Another study in which overweight adults received 50 x 10^9^ CFU/dose of *Bifidobacterium* breve B-3 daily for 12 weeks showed that body fat mass (BFM) decreased and blood parameters improved, and these are related to biomarkers of liver function and inflammation, such as c-glutamyltranspeptidase and high sensitivity C reactive protein (hCRP) (75).

### Interventions related to nutritional state

Malnutrition predisposes individuals to cognitive frailty and vascular risk (76). In addition to nutrition, vascular risk factors could potentially influence cognitive problems related to frailty syndrome (42). This syndrome involves the deficiency of all micronutrients (77), and its risk is directly proportional to concentrations of micronutrients below normal levels (78). Low concentrations of micronutrients are related to frailty as well as prefrailty; this highlights that micronutrients represent a potentially modifiable factor (79). Deficiency of nutrients such as flavonoids, carotenoids, vitamins, n3 fatty acids, and antioxidants promotes inflammation (80). Moreover, the accumulation of reactive oxidation species and nitrogen reactive species leads to cognitive decline (80). Hence, the consumption of dietary antioxidants may offer some benefits because they can eliminate free radicals and decrease oxidative stress.

A poor diet quality and low consumption of vegetable protein can increase the risk of frailty in men and women between 70 and 81 years of age (81). Low-grade inflammation is also present in patients with malnutrition and sarcopenia, and both could be treated with interventions, including protein and energy intake, that can reverse or prevent physio-pathological outcomes (82). A multimodal intervention including exercise and diet optimization can help to prevent frailty syndrome and sarcopenia (83). Nevertheless, the impact of protein dietary interventions is not yet clearly understood.

Another type of intervention involves protein supplementation, followed by a muscular strength exercise program. These interventions could promote muscle mass and strength gain, improve physical performance, and decrease morbidity in older adults at risk of sarcopenia and frailty (84). Supplementation with vitamin D and leucine with other components, such as fiber and minerals, attenuates the progression of low-grade chronic inflammation in older adults with sarcopenia and mobility limitations (85). Furthermore, creatine supplementation is used to treat muscle mass and functionality loss, but the results are inconsistent (86), which may be due to a patient's health status and their habitual diet. Despite their potential use to treat frailty syndrome, these interventions do not directly exert an impact on the gut microbiota.

## Discussion

In this review, we gained a better understanding of concepts about aging, especially about frailty syndrome. As aging is a complex process, numerous authors have pursued a valid definition that is capable of explaining and delimiting it. However, the main purpose of understanding these concepts is to apply them to design better therapeutic strategies for treating frailty syndrome.

Alterations in body composition, cognitive impairment, and neuroendocrine changes have a strong relationship with this disorder. However, how the gut microbiota is associated with these conditions remains unclear and must be further studied. Aging-related diseases have multifactorial causes, but inflammaging has been identified as a key physiopathological mechanism. The gut microbiota actively participates in this process, for instance, in the development of immunological tolerance as well as the synthesis of metabolites in various processes (Di Sabatino et al., 2018, Rinninella et al., 2019, Pascale et al., 2018).

Advances in this field are limited because they are relatively new, and most studies have focused on the brain-intestine axis in animal models and not in humans (42). For example, the brain-gut-microbiota axis is involved in the development of dementia in mouse models (Sadler et al., 2017, Shen et al., 2017, Scott et al., 2017). More translational and clinical research needs to be conducted to understand the role of the gut microbiota composition in the health of older adults and frailty syndrome.

Research on the gut microbiota usually focuses on sequencing 16S rRNA gene amplicons (macromolecules used in bacterial phylogeny and taxonomy), not considering other genes or microorganisms, such as yeasts and viruses, that may be relevant in characterizing microbiota. This may be a limitation in understanding the impact of the gut microbiota on health, and it could represent an area of opportunity to develop future research.

Older adults must be evaluated in a context in which modifications of the gut microbiota are considered possible physio-pathological mechanisms and indeed considered a possible target of treatment or even prophylaxis. To date, interventions on the gut microbiota in patients with frailty syndrome have not been formally considered. The international consensus including European and American institutions that was carried out in 2013 recognized some interventions, but the use of prebiotics and probiotics has not been included in their guidelines. The effects of prebiotics on the gut microbiota of patients with frailty syndrome are unclear and must be considered in current and future investigations.

Despite the fact that there is evidence about the role of gut microbiota in frailty syndrome, there are few clinical studies that demonstrate the benefits of using prebiotics as part of the treatment strategies. For instance, inulin supplementation decreases some frailty signs (Theou et al., 2019, Abizanda et al., 2015). The role of prebiotics in pro- and anti-inflammatory cytokine levels is ambiguous because some of them may either decrease or increase (66). In addition, the reduction of inflammatory factors such as IL17A, IL6, TNF, IFN, IL10, IL8, CD4, CD8, C reactive protein, CXCL11, CCL11, and prebiotic supplementation may not have an association with modifications in the microbiota of frail patients (91). Therefore, further investigations are required to clarify these aspects.

There is also another alternative intervention in which the use of symbiotics has barely been explored. The synergistic action enhances microorganism growth and promotes strain survival (92). Research has evaluated its efficacy mainly in preventing diarrhea associated with antibiotics; moreover, it may be a useful tool to improve the gut microbiota and the health status of older adults (93).

The recent COVID-19 pandemic deserves consideration, as we know inflammaging and frailty lead to poor physical and immune responses. Although probiotics must be carefully administered to critically ill patients, their use may have a benefit when related to SARS-CoV-2 infection (94). Oral administration of probiotics seems to contribute to a better response against viral infection by stimulating the immune response from the gut (95). These findings are relevant; patients with frailty syndrome may show better immune responses through the administration of probiotics or prebiotics and become more able to respond to infections.

To improve the understanding of the modulation of the gut microbiota to treat or prevent frailty syndrome, better comprehension of how the axes of the intestinal microbiota with other specific organs or tissues work during aging is required. Additionally, it is necessary to know which metabolites, neurotransmitters, hormones, or other types of molecular markers participate in these axes. Treatment outcomes, especially in older adults, are often related to life expectancy. Maximum life expectancy and average life expectancy are two parameters considered in assessing aging or senescence, and they are often used as interchangeable terms. Nevertheless, the quality of life in older adults should be given greater attention in aging studies, since a decrease in morbidity is required to consider healthy aging.

## Conclusions

Frailty is related to a high risk of falls, disability, hospitalization, and increased mortality. Moreover, the intestinal microbiota plays a crucial role in health as it is involved in metabolic processes that impact cognition, body composition, and immune function, which are essential to achieve healthy aging. The maintenance of healthy gut microbiota throughout the lifespan could be achieved by following an adequate diet and lifestyle. Moreover, timely interventions for older adults must be conducted to improve their gut microbiota composition during the aging process. These interventions could include probiotic and prebiotic supplementation. Future research must be conducted to elucidate the mechanisms behind the relationship between neurodegeneration and the gut microbiota to improve the diagnosis and therapeutic strategies of frailty syndrome.

## References

[bib1] Di Sabatino A, Lenti MV, Cammalleri L, Corazza GR, Pilotto A (2018). Frailty and the gut. Dig Liver Dis.

[bib2] Bibbo S, Ianiro G, Giorgio V, Scaldaferri F, Masucci L, Gasbarrini A, Cammarota G (2016). The role of diet on gut microbiota composition. Eur Rev Med Pharmacol Sci.

[bib3] Rinninella E, Raoul P, Cintoni M, Franceschi F, Miggiano GAD, Gasbarrini A, Mele MC (2019). What is the Healthy Gut Microbiota Composition? A Changing Ecosystem across Age, Environment, Diet, and Diseases. Microorganisms.

[bib4] Hasan N, Yang H (2019). Factors affecting the composition of the gut microbiota, and its modulation. PeerJ.

[bib5] Balcombe NR (2001). Ageing: definitions, mechanisms and the magnitude of the problem. Best practice & research Clinical Gastroenterology.

[bib6] Cannon ML (2015). What is aging?. Dis Mon.

[bib7] Flatt T (2012). A new definition of aging?. Front Genet.

[bib8] World Health Orgatization Ageing and health. https://www.who.int/news-room/fact-sheets/detail/ageing-and-health. Accessed April, 20222021.

[bib9] Bird ML, Pittaway JK, Cuisick I, Rattray M, Ahuja KD (2013). Age-related changes in physical fall risk factors: results from a 3 year follow-up of community dwelling older adults in Tasmania, Australia. Int J Environ Res Public Health.

[bib10] Carlson C, Merel SE, Yukawa M (2015). Geriatric syndromes and geriatric assessment for the generalist. Med Clin North Am.

[bib11] Morley JE (2016). Frailty and Sarcopenia: The New Geriatric Giants. Rev Invest Clin.

[bib12] Rodriguez-Manas L, Fried LP (2015). Frailty in the clinical scenario. Lancet.

[bib13] Kuzuya M (2019). Era of geriatric medical challenges: Multimorbidity among older patients. Geriatr Gerontol Int.

[bib14] Dent E, Kowal P, Hoogendijk EO (2016). Frailty measurement in research and clinical practice: A review. Eur J Intern Med.

[bib15] Franceschi C, Garagnani P, Vitale G, Capri M, Salvioli S (2017). Inflammaging and 'Garb-aging'. Trends Endocrinol Metab.

[bib16] Hill C, Guarner F, Reid G, Gibson GR, Merenstein DJ, Pot B, Morelli L, Canani RB, Flint HJ, Salminen S (2014). Expert consensus document. The International Scientific Association for Probiotics and Prebiotics consensus statement on the scope and appropriate use of the term probiotic. Nat Rev Gastroenterol Hepatol.

[bib17] Hoffmann DE, Fraser CM, Palumbo FB, Ravel J, Rothenberg K, Rowthorn V, Schwartz J (2013). Science and regulation. Probiotics: finding the right regulatory balance. Science.

[bib18] Reid G, Younes JA, Van der Mei HC, Gloor GB, Knight R, Busscher HJ (2011). Microbiota restoration: natural and supplemented recovery of human microbial communities. Nat Rev Microbiol.

[bib19] Theou O, Jayanama K, Fernandez-Garrido J, Buigues C, Pruimboom L, Hoogland AJ, Navarro-Martinez R, Rockwood K, Cauli O (2019). Can a Prebiotic Formulation Reduce Frailty Levels in Older People?. J Frailty Aging.

[bib20] Gibson GR, Hutkins R, Sanders ME, Prescott SL, Reimer RA, Salminen SJ, Scott K, Stanton C, Swanson KS, Cani PD (2017). Expert consensus document: The International Scientific Association for Probiotics and Prebiotics (ISAPP) consensus statement on the definition and scope of prebiotics. Nat Rev Gastroenterol Hepatol.

[bib21] Gibson GR, Roberfroid MB (1995). Dietary modulation of the human colonic microbiota: introducing the concept of prebiotics. J Nutr.

[bib22] Swanson KS, Gibson GR, Hutkins R, Reimer RA, Reid G, Verbeke K, Scott KP, Holscher HD, Azad MB, Delzenne NM (2020). The International Scientific Association for Probiotics and Prebiotics (ISAPP) consensus statement on the definition and scope of synbiotics. Nat Rev Gastroenterol Hepatol.

[bib23] Kolida S, Gibson GR (2011). Synbiotics in health and disease. Annu Rev Food Sci Technol.

[bib24] Brown M, Sinacore DR, Binder EF, Kohrt WM (2000). Physical and performance measures for the identification of mild to moderate frailty. J Gerontol A Biol Sci Med Sci.

[bib25] Monteiro CA, Cannon G, Levy RB, Moubarac JC, Louzada ML, Rauber F, Khandpur N, Cediel G, Neri D, Martinez-Steele E (2019). Ultra-processed foods: what they are and how to identify them. Public Health Nutr.

[bib26] Chen X, Zhang Z, Yang H, Qiu P, Wang H, Wang F, Zhao Q, Fang J, Nie J (2020). Consumption of ultra-processed foods and health outcomes: a systematic review of epidemiological studies. Nutr J.

[bib27] Arumugam M, Raes J, Pelletier E, Le Paslier D, Yamada T, Mende DR, Fernandes GR, Tap J, Bruls T, Batto JM (2011). Enterotypes of the human gut microbiome. Nature.

[bib28] Biagi E, Franceschi C, Rampelli S, Severgnini M, Ostan R, Turroni S, Consolandi C, Quercia S, Scurti M, Monti D (2016). Gut Microbiota and Extreme Longevity. Curr Biol.

[bib29] Chang CS, Kao CY (2019). Current understanding of the gut microbiota shaping mechanisms. J Biomed Sci.

[bib30] O'Toole PW, Jeffery IB (2015). Gut microbiota and aging. Science.

[bib31] O'Toole PW, Jeffery IB (2018). Microbiome-health interactions in older people. Cell Mol Life Sci.

[bib32] Ticinesi A, Nouvenne A, Tana C, Prati B, Cerundolo N, Miraglia C, De'Angelis GL, Di Mario F, Meschi T (2018). The impact of intestinal microbiota on bio-medical research: definitions, techniques and physiology of a “new frontier”. Acta Biomed.

[bib33] Ticinesi A, Nouvenne A, Cerundolo N, Catania P, Prati B, Tana C, Meschi T (2019). Gut Microbiota, Muscle Mass and Function in Aging: A Focus on Physical Frailty and Sarcopenia. Nutrients.

[bib34] Pascale A, Marchesi N, Marelli C, Coppola A, Luzi L, Govoni S, Giustina A, Gazzaruso C (2018). Microbiota and metabolic diseases. Endocrine.

[bib35] Schonfeld P, Wojtczak L (2016). Short- and medium-chain fatty acids in energy metabolism: the cellular perspective. J Lipid Res.

[bib36] Fung TC, Olson CA, Hsiao EY (2017). Interactions between the microbiota, immune and nervous systems in health and disease. Nat Neurosci.

[bib37] Arpaia N, Campbell C, Fan X, Dikiy S, van der Veeken J, deRoos P, Liu H, Cross JR, Pfeffer K, Coffer PJ (2013). Metabolites produced by commensal bacteria promote peripheral regulatory T-cell generation. Nature.

[bib38] Erny D, Hrabe de Angelis AL, Jaitin D, Wieghofer P, Staszewski O, David E, Keren-Shaul H, Mahlakoiv T, Jakobshagen K, Buch T (2015). Host microbiota constantly control maturation and function of microglia in the CNS. Nat Neurosci.

[bib39] Komanduri M, Gondalia S, Scholey A, Stough C (2019). The microbiome and cognitive aging: a review of mechanisms. Psychopharmacology (Berl).

[bib40] Xu Y, Wang Y, Li H, Dai Y, Chen D, Wang M, Jiang X, Huang Z, Yu H, Huang J (2021). Altered Fecal Microbiota Composition in Older Adults With Frailty. Front Cell Infect Microbiol.

[bib41] Ticinesi A, Tana C, Nouvenne A (2019). The intestinal microbiome and its relevance for functionality in older persons. Curr Opin Clin Nutr Metab Care.

[bib42] Ticinesi A, Tana C, Nouvenne A, Prati B, Lauretani F, Meschi T (2018). Gut microbiota, cognitive frailty and dementia in older individuals: a systematic review. Clin Interv Aging.

[bib43] Verdi S, Jackson MA, Beaumont M, Bowyer RCE, Bell JT, Spector TD, Steves CJ (2018). An Investigation Into Physical Frailty as a Link Between the Gut Microbiome and Cognitive Health. Front Aging Neurosci.

[bib44] Bajaj JS, Ahluwalia V, Steinberg JL, Hobgood S, Boling PA, Godschalk M, Habib S, White MB, Fagan A, Gavis EA (2016). Elderly patients have an altered gut-brain axis regardless of the presence of cirrhosis. Sci Rep.

[bib45] Casati M, Ferri E, Azzolino D, Cesari M, Arosio B (2019). Gut microbiota and physical frailty through the mediation of sarcopenia. Exp Gerontol.

[bib46] Baylis D, Bartlett DB, Syddall HE, Ntani G, Gale CR, Cooper C, Lord JM, Sayer AA (2013). Immune-endocrine biomarkers as predictors of frailty and mortality: a 10-year longitudinal study in community-dwelling older people. Age (Dordr).

[bib47] Gardner M, Lightman S, Kuh D, Comijs H, Deeg D, Gallacher J, Geoffroy MC, Kivimaki M, Kumari M, Power C (2019). Dysregulation of the hypothalamic pituitary adrenal (HPA) axis and cognitive capability at older ages: individual participant meta-analysis of five cohorts. Sci Rep.

[bib48] Le NP, Varadhan R, Fried LP, Cappola AR (2021). Cortisol and Dehydroepiandrosterone Response to Adrenocorticotropic Hormone and Frailty in Older Women. J Gerontol A Biol Sci Med Sci.

[bib49] Marcos-Perez D, Sanchez-Flores M, Maseda A, Lorenzo-Lopez L, Millan-Calenti JC, Pasaro E, Laffon B, Valdiglesias V (2019). Serum cortisol but not oxidative stress biomarkers are related to frailty: results of a cross-sectional study in Spanish older adults. J Toxicol Environ Health A.

[bib53] Keskitalo A, Aatsinki AK, Kortesluoma S, Pelto J, Korhonen L, Lahti L, Lukkarinen M, Munukka E, Karlsson H, Karlsson L (2021). Gut microbiota diversity but not composition is related to saliva cortisol stress response at the age of 2.5 months. Stress.

[bib54] Vagnerova K, Vodicka M, Hermanova P, Ergang P, Srutkova D, Klusonova P, Balounova K, Hudcovic T, Pacha J (2019). Interactions Between Gut Microbiota and Acute Restraint Stress in Peripheral Structures of the Hypothalamic-Pituitary-Adrenal Axis and the Intestine of Male Mice. Front Immunol.

[bib55] Ni Lochlainn M, Bowyer RCE, Steves CJ (2018). Dietary Protein and Muscle in Aging People: The Potential Role of the Gut Microbiome. Nutrients.

[bib56] Ticinesi A, Lauretani F, Milani C, Nouvenne A, Tana C, Del Rio D, Maggio M, Ventura M, Meschi T (2017). Aging Gut Microbiota at the Cross-Road between Nutrition, Physical Frailty, and Sarcopenia: Is There a Gut-Muscle Axis?. Nutrients.

[bib57] Bischoff SC, Boirie Y, Cederholm T, Chourdakis M, Cuerda C, Delzenne NM, Deutz NE, Fouque D, Genton L, Gil C (2017). Towards a multidisciplinary approach to understand and manage obesity and related diseases. Clin Nutr.

[bib58] Backhed F, Ding H, Wang T, Hooper LV, Koh GY, Nagy A, Semenkovich CF, Gordon JI (2004). The gut microbiota as an environmental factor that regulates fat storage. Proc Natl Acad Sci U S A.

[bib59] Poggiogalle E, Lubrano C, Gnessi L, Mariani S, Di Martino M, Catalano C, Lenzi A, Donini LM (2019). The decline in muscle strength and muscle quality in relation to metabolic derangements in adult women with obesity. Clin Nutr.

[bib60] Sachs S, Zarini S, Kahn DE, Harrison KA, Perreault L, Phang T, Newsom SA, Strauss A, Kerege A, Schoen JA (2019). Intermuscular adipose tissue directly modulates skeletal muscle insulin sensitivity in humans. Am J Physiol Endocrinol Metab.

[bib61] van de Wouw M, Schellekens H, Dinan TG, Cryan JF (2017). Microbiota-Gut-Brain Axis: Modulator of Host Metabolism and Appetite. J Nutr.

[bib62] Fetissov SO (2017). Role of the gut microbiota in host appetite control: bacterial growth to animal feeding behaviour. Nat Rev Endocrinol.

[bib63] Tu Y, Yang R, Xu X, Zhou X (2021). The microbiota-gut-bone axis and bone health. J Leukoc Biol.

[bib64] SY Parladore, B Andressa, FR Luiz (2020). The role of Short-Chain Fatty Acids from Gut Microbiota in Gut-Brain Communication. Front Endocrinol (Lausanne).

[bib65] Buigues C, Fernandez-Garrido C, Pruimboom L, Hoogland AJ, Navarro-Martinez R, Martinez-Martinez M, Verdejo Y, Mascaros MC, Peris C, Cauli O (2016). Effect of a Prebiotic Formulation on Frailty Syndrome: A Randomized, Double-Blind Clinical Trial. Int J Mol Sci.

[bib66] Khangwal I, Shukla P (2019). Potential prebiotics and their transmission mechanisms: Recent approaches. J Food Drug Anal.

[bib67] Holscher HD, Bauer LL, Gourineni V, Pelkman CL, Fahey GC, Swanson KS (2015). Agave Inulin Supplementation Affects the Fecal Microbiota of Healthy Adults Participating in a Randomized, Double-Blind, Placebo-Controlled, Crossover Trial. J Nutr.

[bib68] Ampatzoglou A, Atwal KK, Maidens CM, Williams CL, Ross AB, Thielecke F, Jonnalagadda SS, Kennedy OB, Yaqoob P (2015). Increased whole grain consumption does not affect blood biochemistry, body composition, or gut microbiology in healthy, low-habitual whole grain consumers. J Nutr.

[bib69] Salonen A, Lahti L, Salojarvi J, Holtrop G, Korpela K, Duncan SH, Date P, Farquharson F, Johnstone AM, Lobley GE (2014). Impact of diet and individual variation on intestinal microbiota composition and fermentation products in obese men. ISME J.

[bib70] Whisner CM, Martin BR, Nakatsu CH, Story JA, MacDonald-Clarke CJ, McCabe LD, McCabe GP, Weaver CM (2016). Soluble Corn Fiber Increases Calcium Absorption Associated with Shifts in the Gut Microbiome: A Randomized Dose-Response Trial in Free-Living Pubertal Females. J Nutr.

[bib71] Tsai YL, Lin TL, Chang CJ, Wu TR, Lai WF, Lu CC, Lai HC (2019). Probiotics, prebiotics and amelioration of diseases. J Biomed Sci.

[bib72] Brussow H (2019). Probiotics and prebiotics in clinical tests: an update. F1000Res.

[bib73] Leber B, Tripolt NJ, Blattl D, Eder M, Wascher TC, Pieber TR, Stauber R, Sourij H, Oettl K, Stadlbauer V (2012). The influence of probiotic supplementation on gut permeability in patients with metabolic syndrome: an open label, randomized pilot study. Eur J Clin Nutr.

[bib74] Jung SP, Lee KM, Kang JH, Yun SI, Park HO, Moon Y, Kim JY (2013). Effect of Lactobacillus gasseri BNR17 on Overweight and Obese Adults: A Randomized, Double-Blind Clinical Trial. Korean J Fam Med.

[bib75] Minami J, Kondo S, Yanagisawa N, Odamaki T, Xiao JZ, Abe F, Nakajima S, Hamamoto Y, Saitoh S, Shimoda T (2015). Oral administration of Bifidobacterium breve B-3 modifies metabolic functions in adults with obese tendencies in a randomised controlled trial. J Nutr Sci.

[bib76] Panza F, Lozupone M, Solfrizzi V, Sardone R, Dibello V, Di Lena L, D'Urso F, Stallone R, Petruzzi M, Giannelli G (2018). Different Cognitive Frailty Models and Health- and Cognitive-related Outcomes in Older Age: From Epidemiology to Prevention. J Alzheimers Dis.

[bib77] Michelon E, Blaum C, Semba RD, Xue QL, Ricks MO, Fried LP (2006). Vitamin and carotenoid status in older women: associations with the frailty syndrome. J Gerontol A Biol Sci Med Sci.

[bib78] Semba RD, Bartali B, Zhou J, Blaum C, Ko CW, Fried LP (2006). Low serum micronutrient concentrations predict frailty among older women living in the community. J Gerontol A Biol Sci Med Sci.

[bib79] O'Halloran AM, Laird EJ, Feeney J, Healy M, Moran R, Beatty S, Nolan JM, Molloy AM, Kenny RA (2020). Circulating Micronutrient Biomarkers Are Associated With 3 Measures of Frailty: Evidence From the Irish Longitudinal Study on Ageing. J Am Med Dir Assoc.

[bib80] Gomez-Gomez ME, Zapico SC (2019). Frailty, Cognitive Decline, Neurodegenerative Diseases and Nutrition Interventions. Int J Mol Sci.

[bib81] Hengeveld LM, Wijnhoven HAH, Olthof MR, Brouwer IA, Simonsick EM, Kritchevsky SB, Houston DK, Newman AB, Visser M (2019). Prospective Associations of Diet Quality With Incident Frailty in Older Adults: The Health, Aging, and Body Composition Study. J Am Geriatr Soc.

[bib82] Sieber CC (2019). Malnutrition and sarcopenia. Aging Clin Exp Res.

[bib83] Nascimento CM, Ingles M, Salvador-Pascual A, Cominetti MR, Gomez-Cabrera MC, Vina J (2019). Sarcopenia, frailty and their prevention by exercise. Free Radic Biol Med.

[bib84] Liao CD, Chen HC, Huang SW, Liou TH (2019). The Role of Muscle Mass Gain Following Protein Supplementation Plus Exercise Therapy in Older Adults with Sarcopenia and Frailty Risks: A Systematic Review and Meta-Regression Analysis of Randomized Trials. Nutrients.

[bib85] Liberman K, Njemini R, Luiking Y, Forti LN, Verlaan S, Bauer JM, Memelink R, Brandt K, Donini LM, Maggio M (2019). Thirteen weeks of supplementation of vitamin D and leucine-enriched whey protein nutritional supplement attenuates chronic low-grade inflammation in sarcopenic older adults: the PROVIDE study. Aging Clin Exp Res.

[bib86] Candow DG, Forbes SC, Chilibeck PD, Cornish SM, Antonio J, Kreider RB (2019). Variables Influencing the Effectiveness of Creatine Supplementation as a Therapeutic Intervention for Sarcopenia. Front Nutr.

[bib87] Sadler R, Singh V, Benakis C, Garzetti D, Brea D, Stecher B, Anrather J, Liesz A (2017). Microbiota differences between commercial breeders impacts the post-stroke immune response. Brain Behav Immun.

[bib88] Shen L, Liu L, Ji HF (2017). Alzheimer's Disease Histological and Behavioral Manifestations in Transgenic Mice Correlate with Specific Gut Microbiome State. J Alzheimers Dis.

[bib89] Scott KA, Ida M, Peterson VL, Prenderville JA, Moloney GM, Izumo T, Murphy K, Murphy A, Ross RP, Stanton C (2017). Revisiting Metchnikoff: Age-related alterations in microbiota-gut-brain axis in the mouse. Brain Behav Immun.

[bib90] Abizanda P, Lopez MD, Garcia VP, Estrella J D D, da Silva Gonzalez A, Vilardell NB, Torres KA (2015). Effects of an Oral Nutritional Supplementation Plus Physical Exercise Intervention on the Physical Function, Nutritional Status, and Quality of Life in Frail Institutionalized Older Adults: The ACTIVNES Study. J Am Med Dir Assoc.

[bib91] Tran TTT, Cousin FJ, Lynch DB, Menon R, Brulc J, Brown JR, O'Herlihy E, Butto LF, Power K, Jeffery IB (2019). Prebiotic supplementation in frail older people affects specific gut microbiota taxa but not global diversity. Microbiome.

[bib92] Markowiak P, Slizewska K (2017). Effects of Probiotics, Prebiotics, and Synbiotics on Human Health. Nutrients.

[bib93] Olveira G, Gonzalez-Molero I (2016). An update on probiotics, prebiotics and symbiotics in clinical nutrition. Endocrinol Nutr.

[bib94] Padilha de Lima A, Macedo Rogero M, Araujo Viel T, Garay-Malpartida HM, Aprahamian I, Lima Ribeiro SM (2022). Interplay between Inflammaging, Frailty and Nutrition in Covid-19: Preventive and Adjuvant Treatment Perspectives. J Nutr Health Aging.

[bib95] Baud D, Dimoou Agri V, Gibson GR, Reid G, Giannoni E (2020). Using Probiotics to Flatten the Curve of Coronavirus Disease COVID-2019 Pandemic. Front Public Health.

